# Causes of death in people living with HIV: Lessons from five health facilities in Eswatini

**DOI:** 10.4102/sajhivmed.v25i1.1614

**Published:** 2024-10-28

**Authors:** Yves Mafulu, Sukoluhle Khumalo, Victor Williams, Sandile Ndabezitha, Elisha Nyandoro, Nkosana Ndlovu, Alexander Kay, Khetsiwe Maseko, Hlobsile Simelane, Siphesihle Gwebu, Normusa Musarapasi, Arnold Mafukidze, Pido Bongomin, Nduduzo Dube, Lydia Buzaalirwa, Nkululeko Dube, Samson Haumba

**Affiliations:** 1 Department of Care and Treatment, AIDS Healthcare Foundation, Manzini, Eswatini; 2 Center for Global Health Practice and Impact, Georgetown University, Mbabane, Eswatini; 3 Department of Global Health, Julius Center for Health Sciences and Primary Care, University Medical Center, Utrecht University, Utrecht, the Netherlands; 4 Baylor College of Medicine Children’s Foundation, Mbabane, Eswatini; 5 AIDS Healthcare Foundation Africa Bureau, Kampala, Uganda; 6 Center for Global Health Practice and Impact, Georgetown University Medical Center, Washington DC, Washington, United States of America

**Keywords:** HIV, advanced HIV disease, people living with HIV, antiretroviral therapy, Eswatini, tuberculosis, non-communicable disease, COVID-19, malignancies, cervical cancer

## Abstract

**Background:**

Eswatini has a high HIV prevalence in adults and, despite being one of the first countries to achieve the UNAIDS 95-95-95 targets, AIDS-related deaths are still high.

**Objectives:**

This study describes the causes of death among people living with HIV (PLHIV) receiving care at five clinics in Eswatini.

**Method:**

A cross-sectional review of sociodemographic, clinical and mortality data of deceased clients who received care from 01 January 2021 to 30 June 2022, was conducted. Data were extracted from the deceased clients’ clinical records, and descriptive and comparative analysis was performed.

**Results:**

Of 257 clients, 52.5% (*n* = 135) were male, and the median age was 47 years (interquartile range [IQR]: 38, 59). The leading causes of death were non-communicable diseases (NCDs) (*n* = 59, 23.0%), malignancies (*n* = 37, 14.4%), COVID-19 (*n* = 36, 14.0%), and advanced HIV disease (AHD) (*n* = 24, 9.3%). Clients who had been on antiretroviral therapy (ART) for 12–60 months (OR: 0.01; 95% confidence interval [CI]: 0.0006, 0.06) and > 60 months (OR: 0.006; 95% CI: 0.0003, 0.029) had lower odds of death from AHD compared to those on ART for < 12 months. Clients aged ≥ 40 years had higher odds of dying from COVID-19, while female clients (OR: 2.64; 95% CI: 1.29, 5.70) had higher odds of death from malignancy.

**Conclusion:**

Most clients who died were aged 40 years and above and died from NCD-related causes, indicating a need to integrate prevention, screening, and treatment of NCDs into HIV services. Specific interventions targeting younger PLHIV will limit their risk for AHD.

**What this study adds:** People receiving treatment for HIV now live longer and mostly die from non-HIV-related causes, specifically non-communicable diseases. This indicates a need to integrate non-communicable disease screening and care into HIV services.

## Introduction

Global trends in AIDS-related mortality reveal the remarkable success of antiretroviral therapy (ART). HIV-related deaths have decreased from over 2 million in 2004 to 630 000 in 2022, representing 69% fewer deaths.^1,2^ The annual number of deaths among people living with HIV (PLHIV) has fallen substantially, and modelling suggests that the life expectancy of PLHIV is approaching that of the general population.^3^ The improving life expectancy of PLHIV suggests the need for greater attention to the prevention of comorbidities which contribute to higher mortality.^4^ Ageing with HIV presents a new set of challenges due to a combination of age-associated degenerative diseases and comorbidities.^5^

In Africa, HIV/AIDS-related deaths have reduced from over 1 million in 2000 to 380 000 in 2022, primarily due to the introduction of ART.^2,6^ Although HIV/AIDS as a cause of death has dropped from 8th to 19th position globally between 2000 and 2019,^7^ it remains a public health threat in low- and middle-income countries.^7,8^ Studies from sub-Saharan Africa indicate there is a shift in the predominant causes of death from AIDS-related to non-AIDS-defining conditions.^9,10,11^

Eswatini has the highest global prevalence of HIV, at 24.8% in people aged 15 years and above, with an estimated 230 000 PLHIV as of 2023.^1,12^ The country is an early adopter of evidence-based HIV control interventions, having introduced and scaled up a national ART Program in 2004, with the implementation of universal treatment for all in 2016. This has contributed to a 50% decline in HIV-related mortality between 2010 and 2016.^13,14^ As of 2021, AIDS-related deaths in Eswatini had dropped to 225 per 100 000 population from 513 per 100 000 in 2010,^15^ and current projections indicate a continued reduction in mortality.^16^ Eswatini is one of the first countries to achieve the UNAIDS 95-95-95 targets alongside Switzerland, and is on track to achieve HIV epidemic control.^17,18^

With the achievement of the UNAIDS 95-95-95 targets,^17,19^ there is a need for the HIV care and treatment programme in Eswatini to focus on providing a better quality of life for PLHIV by addressing the prevalent causes of morbidity and mortality. To understand the causes of mortality in this population with high ART coverage and HIV viral load (VL) suppression rates, the study aims to describe the specific causes of death amongst PLHIV on treatment at five (AIDS Healthcare Foundation [AHF]-Eswatini) clinics from January 2021 to June 2022.

## Research methods and design

### Study setting and population

This study was conducted at five clinics in Eswatini (AHF Lamvelase Help Centre, AHF Matsapha, AHF Mbabane, AHF Nhlangano, and AHF Piggs Peak). The clinics commenced services in 2007 and, as of December 2023, served about 34 000 PLHIV receiving HIV care and treatment in Eswatini.

Individuals were included in this study if they had been actively receiving HIV care at any of the five clinics in Eswatini and became deceased, with their death being officially recorded from 01 January 2021 to 30 June 2022. Individuals were excluded if their deaths were reported before or after the study period. Formal sample size analysis and sampling were not done.

### Study design and data management

A cross-sectional analysis of mortality data was conducted from the clinics’ electronic database (Antiretroviral Therapy Patient Monitoring and Reporting – APMR), clients’ files, and records of PLHIV who were receiving HIV care and whose death was reported between January 2021 and June 2022.

Data were extracted from the APMR and clients’ chronic care files into an Excel spreadsheet (Online Appendix 1 Table 1) for mortality reporting and audit. The APMR system collects mandatory data related to routine client monitoring, clinical treatment, and outcomes, and mortality data are routinely reported and audited in an Excel spreadsheet. The causes of death are determined by death certificates, declarations, and a combination of information from verbal autopsies (family members or friends), the last morbid conditions, or health information in the medical records. A clinician (senior medical officer or the senior nurse) confirmed the cause of death during routine mortality audits. The medical manager validated all mortality data from different health facilities. Death is reported as unknown when all the above sources are not informative. Advanced HIV disease was attributed to PLHIV whose CD4+ cell count was less than 100 cells/µL at the time of their death and/or if they were reported to have died with World Health Organization (WHO) stage 3 or stage 4 conditions.

**TABLE 1:** Characteristics of deceased clients.

Client characteristic	Female (*n* = 122)					Male (*n* = 135)					Total (*N* = 257)				
	*n*	%	Mean ± s.d.	Median	IQR	*n*	%	Mean ± s.d.	Median	IQR	*n*	%	Mean ± s.d.	Median	IQR
**Clinic**															
Lamvelase	68	55.74	-	-	-	56	41.48	-	-	-	124	48.25	-	-	-
Matsapha	30	24.59	-	-	-	29	21.48	-	-	-	59	22.96	-	-	-
Mbabane	8	6.56	-	-	-	17	12.59	-	-	-	25	9.73	-	-	-
Nhlangano	15	12.30	-	-	-	30	22.22	-	-	-	45	17.51	-	-	-
Pigg’s Peak	1	0.82	-	-	-	3	2.22	-	-	-	4	1.56	-	-	-
**Age (years)**	-	-	47.25 ± 13.71	47.00	37.00–57.00	-	-	49.70 ± 14.04	48.00	40.00–61.00	-	-	48.54 ± 13.91	47.00	38.00–59.00
≤ 19	0	0.00	-	-	-	2	1.48	-	-	-	2	0.78	-	-	-
20–29	11	9.02	-	-	-	6	4.44	-	-	-	17	6.61	-	-	-
30–39	29	23.77	-	-	-	25	18.52	-	-	-	54	21.01	-	-	-
40–49	29	23.77	-	-	-	38	28.15	-	-	-	67	26.07	-	-	-
50–59	30	24.59	-	-	-	24	17.78	-	-	-	54	21.01	-	-	-
≥ 60	23	18.85	-	-	-	40	29.63	-	-	-	63	24.51	-	-	-
**Functional status**															
Ambulatory	16	13.11	-	-	-	27	20.00	-	-	-	43	16.73	-	-	-
Bedridden	9	7.38	-	-	-	4	2.96	-	-	-	13	5.06	-	-	-
Walking	97	79.51	-	-	-	104	77.04	-	-	-	201	78.21	-	-	-
**Baseline CD4 (cells/μL)**	-	-	247.55 ± 236.72	177.00	72.00–333.00	-	-	218.82 ± 253.85	180.00	63.00–311.00	-	-	231.99 ± 246.00	177.50	70.00–315.50
100–199	25	20.49	-	-	-	22	16.30	-	-	-	47	18.29	-	-	-
200–349	23	18.85	-	-	-	32	23.70	-	-	-	55	21.40	-	-	-
< 100	29	23.77	-	-	-	42	31.11	-	-	-	71	27.63	-	-	-
≥ 350	22	18.03	-	-	-	21	15.56	-	-	-	43	16.73	-	-	-
Not available	23	18.85	-	-	-	18	13.33	-	-	-	41	15.95	-	-	-
**Last CD4 (cells/μL)**	-	-	485.96 ± 291.73	469.50	275.00–640.00	-	-	434.27 ± 297.56	406.50	237.50–538.00	-	-	460.36 ± 295.06	418.50	254.00–598.00
100–199	6	4.92	-	-	-	13	9.63	-	-	-	19	7.39	-	-	-
200–349	19	15.57	-	-	-	17	12.59	-	-	-	36	14.01	-	-	-
< 100	10	8.20	-	-	-	10	7.41	-	-	-	20	7.78	-	-	-
≥ 350	71	58.20	-	-	-	64	47.41	-	-	-	135	52.53	-	-	-
Not available	16	13.11	-	-	-	31	22.96	-	-	-	47	18.29	-	-	-
**ART (No/Yes)**															
No	3	2.46	-	-	-	4	2.96	-	-	-	7	2.72	-	-	-
Yes	119	97.54	-	-	-	131	97.04	-	-	-	250	97.28	-	-	-
**ART regimen**															
ABC/3TC/DTG	11	9.02	-	-	-	12	8.89	-	-	-	23	8.95	-	-	-
ABC/3TC/EFV	1	0.82	-	-	-	2	1.48	-	-	-	3	1.17	-	-	-
ABC/3TC/LPVr	0	0 0.00	-	-	-	1	0.74	-	-	-	1	0.39	-	-	-
AZT/3TC/DTG	5	4.10	-	-	-	7	5.19	-	-	-	12	4.67	-	-	-
AZT/3TC/EFV	1	0.82	-	-	-	0	0.00	-	-	-	1	0.39	-	-	-
TDF/3TC/DTG	74	60.66	-	-	-	103	76.30	-	-	-	177	68.87	-	-	-
TDF/3TC/EFV	27	22.13	-	-	-	7	5.19	-	-	-	34	13.23	-	-	-
Not on ART	3	2.46	-	-	-	3	2.22	-	-	-	6	2.33	-	-	-
**Latest VL**															
No VL	4	3.28	-	-	-	6	4.44	-	-	-	10	3.89	-	-	-
Not eligible	16	13.12	-	-	-	20	14.81	-	-	-	36	14.01	-	-	-
Not suppressed	3	2.46	-	-	-	3	2.22	-	-	-	6	2.33	-	-	-
Suppressed	99	81.15	-	-	-	106	78.52	-	-	-	205	79.77	-	-	-
**Duration on ART (months)**	-	-	84.48 ± 56.72	88.00	37.00–127.00	-	-	73.15 ± 55.13	71.00	24.00–115.00	-	-	78.52 ± 56.07	78.00	29.00 – 120.00
< 12 months	16	13.11	-	-	-	24	17.78	-	-	-	40	15.56	-	-	-
12–60 months	33	27.05	-	-	-	37	27.41	-	-	-	70	27.24	-	-	-
61–120 months	39	31.97	-	-	-	49	36.30	-	-	-	88	34.24	-	-	-
> 120 months	34	27.87	-	-	-	25	18.52	-	-	-	59	22.96	-	-	-
**Last seen by**															
Doctor	34	27.87	-	-	-	31	22.96	-	-	-	65	25.29	-	-	-
Nurse	79	64.75	-	-	-	89	65.93	-	-	-	168	65.37	-	-	-
Unknown	9	7.38	-	-	-	15	11.11	-	-	-	24	9.34	-	-	-
**Baseline WHO stage**															
1	66	54.10	-	-	-	71	52.59	-	-	-	137	53.31	-	-	-
2	25	20.49	-	-	-	18	13.33	-	-	-	43	16.73	-	-	-
3	13	10.66	-	-	-	24	17.78	-	-	-	37	14.40	-	-	-
4	18	14.75	-	-	-	22	16.30	-	-	-	40	15.56	-	-	-

3TC, lamivudine; ABC, abacavir; ART, antiretroviral therapy; AZT, zidovudine; DTG, dolutegravir; EFV, efavirenz; IQR, interquartile range; LPV, lopinavir; s.d., standard deviation; TDF, tenofovir disoproxil fumarate; VL, viral load; WHO, World Health Organization.

The Excel spreadsheets used for data collection are standardised across all AIDS Healthcare Foundation sites in Eswatini. They include the clinic ID (file number), gender, age at the time of death, date of HIV test, date of death (or of notification if the date of death is unknown), WHO stage, functional status at the time of death, ART status, duration in care, duration on ART, initial CD4, last CD4, date of last visit, cause or condition at death, HIV regimen, last VL, and last clinician who attended the client (nurse or doctor). The initial CD4 is the CD4 at ART initiation, and the last CD4 is the last available CD4 in the client’s records.

As of 2018, clients stable on ART with undetectable VL and CD4 count ≥ 350 cells/µL were no longer required to undergo routine CD4 count monitoring,^20^ indicating that the last CD4 could have been documented several years before the client’s demise. The last VL was only considered if taken within the 12 months before the client’s previous visit. However, based on the 2018 Eswatini Integrated HIV Management guidelines, clients were only eligible for VL 6 months after ART initiation. Therefore, all clients without VL in the first 6 months of ART initiation were labelled ‘Not Eligible’.

Using a programmatic approach, two clinicians assigned the reasons for death to nine different categories: Accident or Assault, Advanced HIV disease (AHD) conditions, COVID-19 Malignancy, Non-Communicable diseases (NCDs), Organ failure, Suicide, Tuberculosis, Others, and Unknown. In the absence of a CD4 of < 200 cells/µL and WHO stage 3 or stage 4 conditions or any other cause of death, clients were considered to have died of an AHD if they were enrolled in care within 6 months before their death, which coincided with a positive HIV test or ART initiation. Malignancies, which are also NCDs, were classified separately, to highlight the importance of malignancies as a cause of death among PLHIV. At a programmatic level, specific interventions targeting AIDS-related malignancies can be prompted by these statistics. Clients deceased with tuberculosis were grouped separately even if comorbidities or CD4 level would have classified them otherwise (e.g. AHD); we used the same approach for COVID-19. All other causes of death that were in numbers too small to constitute a category were grouped as ‘Others’. Functional status was defined as walking, when the client can carry out regular work in or outside the home, harvest crops, attend school, or, for children, engage in everyday activities or play; ambulatory, when the client can carry out daily activities but is unable to work or play; or bedridden, when the client is unable to carry out daily activities.^21,22^

### Data analysis

The data were initially checked for accuracy, consistency, and completeness. Where discrepancies were observed, the data were verified with the original client mortality record to ensure accuracy and completeness. A descriptive analysis of clinical and sociodemographic characteristics was performed; categorical descriptors were presented as numbers with proportions, while numeric descriptors were presented as a mean with standard deviation (s.d.) or median with interquartile range (IQR). The different causes of death were identified and grouped to identify the overall and cause-specific causes. The prevalence of each cause of death was determined as the number of each cause of death expressed as a proportion of the total number of deceased clients. This was presented using a table and a chart based on the deceased’s clinical and sociodemographic features.

A comparative analysis using Pearson χ^2^ or Fischer’s exact test for categorical variables, *T*-test or Mann-Whitney *U* test for continuous variables was performed to identify associations between causes of death and clinical and sociodemographic characteristics of the deceased. Bivariate regression analysis used R code for variables that showed a statistical difference during the comparative analysis. The odds ratio (OR) was estimated to compare different categories. Statistical significance was set at *P* < 0.05, with a 95% confidence interval (95% CI). Stata version 15 (StataCorp LLC, College Station, Texas, United States) was used for statistical analysis.

### Ethical considerations

The Eswatini Health and Human Research Review Board (EHHRRB) approved this study (reference no.: EHHRRB 120/2023) and provided a waiver for informed consent since we only accessed the clients’ records. Deidentified data were used for analysis to ensure confidentiality.

## Results

### Sociodemographic and clinical characteristics of deceased clients

Table 1 summarises the deceased clients’ characteristics (*n* = 257). The median age was 47 years (IQR: 38, 59). The majority who died (*n* = 240, 92.61%) were aged ≥ 30 years, with more deaths in the 40–49 (*n* = 67, 26%) and ≥ 60 (*n* = 63, 24.51%) years age groups. The median baseline CD4 count for all clients was 177 cells/µL (IQR: 71, 315), while the last CD4 count before death was 418 cells/µL (IQR: 255, 596). Based on the last documented VL result, 80% (*n* = 205) had a suppressed VL (< 1000 copies/mL). Most clients at the time of death (*n* = 250, 97.28%) had commenced ART, and 82.49% (*n* = 212) were on a dolutegravir-based ART regimen. The median duration of ART was 78 months (IQR: 29, 120), and 16% (*n* = 40) had received ART for less than 12 months at the time of death.

### Specific causes of death

Table 2 and Online Appendix 1 Table 2 summarise the specific causes of death (*n* = 209) and those with an unknown cause of death (*n* = 48). The leading causes of death were NCDs (*n* = 59, 22.96%), malignancies (*n* = 37, 14.40%), COVID-19 (*n* = 36, 14.01%) and AHD (*n* = 24, 9.34%). Of the 257, only 7.00% (*n* = 18) died from accident or assault, 4.28% (*n* = 11) from organ failure, 2.72% (*n* = 7) from tuberculosis, and 2.33% (*n* = 6) from suicide. Forty-eight (18.68%) had an unknown cause of death. Most clients who died were from the Lamvelase clinic, mostly from NCD (*n* = 29), malignancy (*n* = 20) and COVID-19 (*n* = 14).

**TABLE 2:** Description of specific causes of death by client’s demographic and clinical characteristics.

Characteristic	AHD (*n* = 24)		Assault or accident (*n* = 18)		COVID-19 (*n* = 36)		Malignancy (*n* = 37)		NCD (*n* = 59)		Organ failure (*n* = 11)		Others (*n* = 11)		Suicide (*n* = 6)		Tuberculosis (*n* = 7)		Unknown (*n* = 48)		Total (*N* = 257)	
	*n*	%	*n*	%	*n*	%	*n*	%	*n*	%	*n*	%	*n*	%	*n*	%	*n*	%	n	%	*n*	%
**Clinic**																						
Lamvelase	6	4.84	7	5.65	14	11.29	20	16.13	29	23.39	3	2.42	6	4.84	3	2.42	3	2.42	33	26.61	124	100.00
Matsapha	4	6.78	3	5.08	9	15.25	9	15.25	15	25.42	6	10.17	4	6.78	2	3.39	2	3.39	5	8.47	59	100.00
Mbabane	3	12.00	2	8.00	7	28.00	6	24.00	5	20.00	0	0.00	1	4.00	0	0.00	1	4.00	0	0.00	25	100.00
Nhlangano	11	24.44	5	11.11	6	13.33	2	4.44	10	22.22	0	0.00	0	0.00	1	2.22	0	0.00	10	22.22	45	100.00
Pigg’s Peak	0	0.00	1	25.00	0	0.00	0	0.00	0	0.00	2	50.00	0	0.00	0	0.00	1	25.00	0	0.00	4	100.00
**Gender**																						
Female	9	7.38	6	4.92	14	11.48	25	20.49	32	26.23	4	3.28	5	4.10	3	2.46	4	3.28	20	16.39	122	100.00
Male	15	11.11	12	8.89	22	16.30	12	8.89	27	20.00	7	5.19	6	4.44	3	2.22	3	2.22	28	20.74	135	100.00
**Age category (years)**																						
< 19	1	50.00	0	0.00	0	0.00	0	0.00	1	50.00	0	0.00	0	0.00	0	0.00	0	0.00	0	0.00	2	100.00
20–29	1	5.88	3	17.65	1	5.88	0	0.00	3	17.65	0	0.00	0	0.00	3	17.65	1	5.88	5	29.41	17	100.00
30–39	10	18.52	5	9.26	2	3.70	7	12.96	13	24.07	3	5.56	6	11.11	2	3.70	1	1.85	5	9.26	54	100.00
40–49	7	10.45	5	7.46	13	19.40	13	19.40	7	10.45	3	4.48	2	2.99	1	1.49	1	1.49	15	22.39	67	100.00
50–59	4	7.41	3	5.56	11	20.37	8	14.81	17	31.48	2	3.70	0	0.00	0	0.00	2	3.70	7	12.96	54	100.00
≥ 60	1	1.59	2	3.17	9	14.29	9	14.29	18	28.57	3	4.76	3	4.76	0	0.00	2	3.17	16	25.40	63	100.00
**WHO stage**																						
1	3	2.19	13	9.49	22	16.06	20	14.60	32	23.36	7	5.11	8	5.84	5	3.65	2	1.46	25	18.25	137	100.00
2	1	2.33	1	2.33	7	16.28	7	16.28	11	25.58	2	4.65	1	2.33	1	2.33	0	0.00	12	27.91	43	100.00
3	6	16.22	4	10.81	5	13.51	2	5.41	5	13.51	1	2.70	2	5.41	0	0.00	4	10.81	8	21.62	37	100.00
4	14	35.00	0	0.00	2	5.00	8	20.00	11	27.50	1	2.50	0	0.00	0	0.00	1	2.50	3	7.50	40	100.00
**Baseline CD4 (cells/μL)**																						
< 100	13	18.31	3	4.23	7	9.86	8	11.27	15	21.13	5	7.04	2	2.82	1	1.41	3	4.23	14	19.72	71	100.00
100–199	1	2.13	3	6.38	9	19.15	7	14.89	11	23.40	3	6.38	1	2.13	0	0.00	0	0.00	12	25.53	47	100.00
200–349	0	0.00	5	9.09	8	14.55	8	14.55	17	30.91	0	0.00	4	7.27	2	3.64	1	1.82	10	18.18	55	100.00
≥ 350	0	0.00	5	11.63	3	6.98	8	18.60	9	20.93	2	4.65	2	4.65	2	4.65	2	4.65	10	23.26	43	100.00
Not available	10	24.39	2	4.88	9	21.95	6	14.63	7	17.07	1	2.44	2	4.88	1	2.44	1	2.44	2	4.88	41	100.00
**ART duration (months)**																						
< 12	22	55.00	1	2.50	3	7.50	0	0.00	5	12.50	1	2.50	0	0.00	0	0.00	2	5.00	6	15.00	40	100.00
12–60	1	1.43	10	14.29	15	21.43	11	15.71	13	18.57	3	4.29	2	2.86	5	7.14	1	1.43	9	12.86	70	100.00
61–120	1	1.14	4	4.55	7	7.95	13	14.77	30	34.09	6	6.82	4	4.55	0	0.00	3	3.41	20	22.73	88	100.00
> 120	0	0.00	3	5.08	11	18.64	13	22.03	11	18.64	1	1.69	5	8.47	1	1.69	1	1.69	13	22.03	59	100.00
**Functional status**																						
Ambulatory	9	20.93	1	2.33	3	6.98	9	20.93	9	20.93	2	4.65	0	0.00	2	4.65	5	11.63	3	6.98	43	100.00
Bedridden	2	15.38	2	15.38	0	0.00	2	15.38	5	38.46	1	7.69	0	0.00	0	0.00	0	0.00	1	7.69	13	100.00
Walking	13	6.47	15	7.46	33	16.42	26	12.94	45	22.39	8	3.98	11	5.47	4	1.99	2	1.00	44	21.89	201	100.00

AHD, advanced HIV disease; ART, antiretroviral therapy; NCD, non-communicable disease; WHO, World Health Organization.

### Non-communicable diseases

The most reported NCD causes of death were cardiovascular diseases (including hypertension and stroke) (*n* = 18), chronic kidney disease (*n* = 15), chronic anaemia of unknown cause (*n* = 6), and diabetes (*n* = 5). More NCD-related deaths (*n* = 32, 54%) occurred in female clients, and while all age groups contributed to NCD deaths, the majority were ≥ 30 years old (*n* = 55, 93%), mainly with baseline WHO stage 1 or stage 2 HIV disease (*n* = 43, 72.88%). Most (*n* = 54, 91.53%) had been on ART for ≥ 12 months.

### Malignancy

The most reported malignancies were that of the cervix and urogenitalia (*n* = 8), prostate and penile tissue (*n* = 4), skin and soft tissues (*n* = 4), breast (*n* = 3) and eye (*n* = 2). More female clients died from malignancies (*n* = 25, 67.56%) than male clients (*n* = 12, 32.43%), and the majority who died were aged ≥ 40 years (*n* = 30, 81%).

### COVID-19

More male (*n* = 22, 61.11%) than female clients (*n* = 14, 38.89%) died from COVID-19-related deaths, and the majority of those who died (*n* = 33, 91.67%) were aged ≥ 40 years old. Twenty-nine (80.56%) were classified as WHO stage 1 or stage 2, and 16 (44.40%) had a baseline CD4 < 200 cells/µL. The majority who died from COVID-19 had been on ART for ≥ 12 months (*n* = 33, 91.67%) and had a walking functional status (*n* = 33, 91.67%).

### Advanced HIV disease

At baseline, 118 clients (45.91%) presented with AHD (CD4 < 200 cells/µL), while only 39 (15.18%) had a last CD4 < 200 cells/µL. The majority who died from AHD-related conditions were male clients (*n* = 15, 62.50%), mostly aged ≥ 30 years (*n* = 22, 91.67%). Most (*n* = 20, 83.33%) were classified as WHO stage 3 or stage 4, with more than half of these (*n* = 14, 58.33%) presenting with a baseline CD4 < 200 cells/µL, and 91.67% (*n* = 22) on ART for less than 12 months.

### Assault- or accident-related deaths

More male clients (*n* = 12, 66.77%) died from assault or accident than female clients (*n* = 6, 33.33%), with a similar distribution across age groups, although most were from the age group 30–49 years (*n* = 10, 55.56%). In addition, 72.22% were classified as WHO stage 1, with a similar number of deaths across the different baseline CD4 groups, and more than half of these had been on ART for 12 to 60 months; 83.33% (*n* = 15) had a walking functional status.

### Organ failure, suicide, tuberculosis, and other causes of death

Organ failure was similar between the sexes and across all age groups after age 29. The majority had been on ART for ≥ 12 months (*n* = 10, 90.91%), and 72.73% (*n* = 8) had a walking functional status. Most who died from suicide were in WHO stage 1 or stage 2, and most had been on ART for 12 to 60 months (*n* = 5, 83.33%). Deaths from tuberculosis were similar across both sexes, and most (*n* = 5, 71%) were classified as WHO stage 3 or stage 4, with varied baseline CD4 and mostly ambulatory functional status (*n* = 5, 71.43%). Causes of death classified as ‘Other’ include pneumonia (with similar chest symptoms) (*n* = 6), acute gastroenteritis (*n* = 2), sudden collapse (*n* = 2), and meningitis (*n* = 1).

### Comparison of deceased characteristics and the four leading causes of death

Table 3 describes the bivariate analysis of the different associations between the clients’ sociodemographic and clinical characteristics and the four leading causes of death. Compared to clients younger than 40 years, older clients were less likely to die from AHD; the odds were lowest for those aged ≥ 60 years (OR: 0.08; 95% CI: 0.004, 0.44). Similarly, clients who had been on ART for 12 to 60 months (OR: 0.01; 95% CI: 0.0006, 0.06) and > 60 months (OR: 0.006; 95% CI: 0.0003, 0.029) had lower odds of death due to AHD compared to those on ART for less than 12 months. Clients with ambulatory functional status (OR: 3.83; 95% CI: 1.48–9.60) had higher odds of death from AHD than those with a walking functional status.

**TABLE 3:** Bivariate analysis of the effect of the different client characteristics on the top four causes of death.

Characteristic	AHD		COVID-19		Malignancy		NCD	
	OR	95% CI	OR	95% CI	OR	95% CI	OR	95% CI
**Sex**								
Male	Ref.	-	Ref.	-	Ref.	-	Ref.	-
Female	0.63	0.26–1.49	0.67	0.32–1.36	2.64**	1.29–5.70**	1.42	0.79–2.56
**Age (years)**								
< 40	Ref.	-	Ref.	-	Ref.	-	Ref.	-
40–49	0.59	0.21–1.58	5.62*	1.71–25.40*	2.27	0.87–6.41	0.38**	0.14–0.96**
50–59	0.41	0.11–1.25	5.97*	1.75–27.49*	1.64	0.55–4.98	1.51	0.68–3.35
≥ 60	0.08**	0.004–0.44**	3.89*	1.10–18.16*	1.57	0.55–4.66	1.31	0.61–2.87
**ART duration (months)**								
< 12	Ref.	-	Ref.	-	Ref.	-	Ref.	-
12–60	0.01**	0.0006–0.06**	3.36**	1.02–15.25**	-	-	1.59	0.55–5.32
> 60	0.006**	0.0003–0.029**	1.72	0.54–7.63	-	-	2.70**	1.07–8.31**
**Baseline CD4 (cells/μL)**								
< 200	Ref.	-	Ref.	-	Ref.	-	Ref.	-
≥ 200	-	-	0.81	0.35–1.81	1.34	0.62–2.89	1.28	0.68–2.39
Not available	2.40	0.95–5.90	1.79	0.70–4.38	1.18	0.39–3.15	0.73	0.27–1.76
**Last CD4 (cells/μL)**								
< 200	Ref.	-	Ref.	-	Ref.	-	Ref.	-
≥ 200	-	-	0.94	0.38–2.70	1.71	0.62–6.06	1.63	0.71–4.26
Unavailable	2.0	0.76–5.56	0.65	0.17–2.36	1.04	0.26–4.49	0.8	0.25–2.56
**Last VL**								
Not suppressed	Ref.	-	Ref.	-	Ref.	-	Ref.	-
Suppressed	-	-	-	-	1.03	0.16–20.0	1.66	0.26–32.1
Not eligible	2.24	0.39–1.76	-	-	-	-	0.63	0.71–13.5
**ART**								
No	Ref.	Ref.	-	Ref.	-	Ref.	-
Yes	0.07	0.01–0.31	-	-	-	-	-	-
**Functional status**								
Walking	Ref.	-	Ref.	-	Ref.	-	Ref.	-
Bedridden	2.63	0.38–11.2	-	-	1.22	0.18–4.90	2.17	0.63–6.82
Ambulatory	3.83**	1.48–9.60**	0.38	0.09–1.14	1.58	0.73–4.03	0.92	0.39–1.99

AHD, advanced HIV disease; ART, antiretroviral therapy; CI, confidence interval; NCD, non-communicable disease; OR, odds ratio; Ref., reference value; VL, viral load.

* , statistically significant at *P* ≤ 0.05;

** , statistically significant at *P* ≤ 0.01.

Clients aged 40–49 years (OR: 5.62; 95% CI: 1.7, 25.4), 50–59 years (OR: 5.97; 95% CI: 1.8, 27.5) and ≥ 60 years (OR: 3.89; 95% CI: 1.10, 18.16) had higher odds of dying from a COVID-19-related complication compared to those aged < 40 years. Female clients (OR: 2.64; 95% CI: 1.29, 5.70) had higher odds of death from a malignancy-related cause than male clients. For NCDs, the age group 40–49 years (OR: 0.38; 95% CI: 0.14, 0.96) had lower odds of death compared to those younger than 40 years, while those on ART for > 60 months (OR: 2.70; 95% CI: 1.07, 8.31) had higher odds of death due to an NCD compared to those on ART for < 12 months.

## Discussion

We aimed to describe the specific causes of death amongst PLHIV who died or whose death was reported from January 2021 to June 2022. NCDs, malignancies, COVID-19 and AHD were the leading causes of death. More than half who died were male, and most were aged ≥ 40 years. NCD-related deaths increased with age and duration of ART. The odds of dying from COVID-19-related complications were higher for those aged ≥ 40 years, and more female clients died from malignancy-related causes than male clients. Older clients were less likely to die from AHD, and those on ART for < 12 months were more likely to die from AHD.

Our finding of NCDs as the leading cause of death amongst PLHIV is not surprising. Earlier studies from Tanzania, Zimbabwe and Uganda, while observing the traditional infectious disease causes of death, such as tuberculosis, AIDS-related opportunistic infections and malignancies, observed increasing trends in NCD as a cause of death from 2015.^9,10,23,24,25^ Cardiovascular diseases (hypertension, stroke), kidney diseases, diabetes mellitus and malignancies have been identified as the most common NCD causes of death from these earlier studies.^9,10,23^ Additionally, those who died from NCDs were observed to be much older, above 40 years.^24,26^ Our analysis identified NCDs as the leading cause of mortality, confirming that these trends have progressed.

Unlike studies conducted before 2018 in Burkina Faso, Uganda, Ghana, and South Africa,^23,24,26,27^ tuberculosis was not among our cohort’s top three causes of death. This could be attributed to the fact that PLHIV now access more effective ART with improved immune response against opportunistic infections, and are living to older ages (common with NCDs).^27,28^ For instance, 80% of clients in our cohort had a suppressed last VL, and 71% were ≥ 40 years old, an age commonly associated with NCDs. In addition to the above, HIV-tuberculosis coinfection has dropped over the years in Eswatini, which could be partially attributed to the high coverage of tuberculosis prophylactic treatment amongst PLHIV in Eswatini.^29^

The observed deaths from malignancies in our cohort, mainly from genital cancers amongst female clients, indicate a limited availability of cancer prevention, screening, and treatment services within the HIV programme in Eswatini. For instance, human papillomavirus (HPV) vaccines have only been recently rolled out and are not widely accessible, and few eligible women living with HIV have access to cervical screening and treatment services, as this is offered in only 37% of health facilities.^30,31^ Previously, most PLHIV died from malignancies caused by non-Hodgkin lymphoma and Kaposi Sarcoma.^32^ Few male clients, however, still die of a malignant condition.

Death from COVID-19-related complications ranked high in our list of causes of death – not surprising, as data from our cohort were obtained at the peak of the COVID-19 pandemic. Evidence suggests that most COVID-19-related deaths occurred in people aged ≥ 40 years,^33^ with an increased risk of death in those with comorbid conditions, including hypertension, diabetes, renal diseases, malignancies, and clients undergoing dialysis.^33^ Given that most of our clients who died had an NCD and were older than 40 years, we believe they were also at risk of COVID-19 and could also have had COVID-19-related complications.

Contrary to earlier studies in Tanzania and Zimbabwe, where ≥ 50% of HIV-related deaths occurred within the first year of commencing ART and from acute causes,^9,10^ most of our clients survived the first year of treatment, with only 15% dying within the first 12 months of treatment and 57% after 60 months of treatment, indicating that clients are more stable at diagnosis. The causes of death have shifted from acute to more chronic conditions. In three earlier studies from Sierra Leone,^34^ Ghana,^35^ and five West African countries,^25^ more deaths were from AHD amongst bedridden inpatients, usually within the first week of admission, primarily due to anaemia, diarrhoeal disease, cerebral toxoplasmosis, or tuberculosis.^25,34,35^ This is different from our study, as only 5% of clients who died were bedridden, with older adults (≥ 40 years) having lower odds of dying from AHD. This observed difference could be due to changes in treatment guidelines, for example test-and-treat,^36^ where clients commence treatment immediately after a positive diagnosis instead of depending on the CD4 count, which made a lot of clients present late with AHD,^3,37^ as well as the introduction of a more potent ART such as dolutegravir and the scale-up of HIV prevention, testing and treatment services in high-burden countries and amongst different sub-population groups.

### Strengths and limitations

Given the widespread use of ART in Eswatini and the achievement of HIV epidemic control, this study contributes to the literature on the possible causes of death in a mature HIV programme. This will guide other programmes to institute adequate preventive measures when scaling up HIV services. Second, data for this study were obtained during the COVID-19 pandemic, which enabled us to explore the associations between different client characteristics and COVID-19 as a cause of death.

However, our study has limitations. First, we used data from health facility records to determine the cause of death without forensics, comprehensive autopsy reports or, at a minimum, verbal autopsy. Some causes of death may have been incorrectly assigned, especially for those who died at home. It was determined that the client’s most recent documented condition was the cause of death when there was no identifiable cause of death. A unified vital records system for reporting and documenting deaths is yet to be rolled out in Eswatini, and our approach provided an approximate method for describing the cause of death. Second, service delivery during the pandemic was variable and could have limited services received by a client. Third, we could not identify the cause of death for about a fifth of the clients, and mortality might have been underestimated due to unreported deaths among unreachable clients reported as lost to follow-up. This could have biased our estimates for the causes of death. Despite these limitations, we believe some key learnings from this study will be helpful to different cadres of healthcare workers and HIV programme implementers.

## Conclusion

As most of our clients were ≥ 40 years old and died from an NCD; the integration of NCD screening and treatment services into infectious disease programmes, as recommended by the WHO.^38^ More women died from malignancies than men, which could have been avoided if HPV vaccines and screening for cervical cancer were widely accessible. Context-specific interventions targeting men and middle-aged people (30–39 years age group) aimed at early diagnoses and treatment can further limit the presentation of AHD and death in middle-aged people. Finally, the rollout of an effective vital records registration system for deaths would help to track the number, trends and causes of death in the general population and amongst ART clients across multiple databases and through records linkage.
